# Fruit Cuticle Thickness and Anatomical Changes in Pedicel Xylem Vessels Influence Fruit Transpiration and Calcium Accumulation in Cranberry Fruit

**DOI:** 10.1111/ppl.70036

**Published:** 2025-01-10

**Authors:** Pedro Rojas‐Barros, Jane Wernow, Beth Ann Workmaster, Juan Zalapa, Jyostna Mura Devi, Amaya Atucha

**Affiliations:** ^1^ Department of Plant and Agroecosystem Sciences University of Wisconsin‐Madison Madison WI; ^2^ USDA‐ARS, Vegetable Crops Research Unit, Department of Plant and Agroecosystem Sciences University of Wisconsin‐Madison Madison WI

## Abstract

Ca is a key nutrient for fruit quality due to its role in bonding with pectin in the cell wall, providing strength through cell‐to‐cell adhesion, thus increasing fruit firmness and extending post‐harvest life. However, Ca accumulation is mostly limited to the initial stages of fruit development due to anatomical and physiological changes that occur as fruits develop. The objective of this study was to evaluate fruit transpiration, cuticle thickness, and pedicel vessel changes during cranberry fruit development and the effect these parameters might have on Ca translocation. ‘Stevens’ cranberry fruits were collected weekly, starting seven days after full bloom (DAFB) until 70 DAFB. For each collection date, fruit transpiration was evaluated in the field, and samples were taken to analyze total fruit Ca content, stomata density, cuticle thickness, pedicel anatomical changes, and xylem functionality. Ca accumulation in the fruit exhibited a sigmoidal curve, beginning at 0.04 mg per berry at 7 DAFB, increasing to a maximum of 0.1 mg per berry at 28 DAFB, and remaining constant until harvest (70 DAFB). Fruit Ca accumulation was mostly explained by fruit transpiration, which exhibited a similar sigmoidal pattern. The rapid decline in fruit transpiration was largely modulated by increases in cuticle thickness, as well as anatomical changes in the pedicel xylem, thereby reducing the capacity to transport water and nutrients into the fruit. Thus, this research could help cranberry growers maximize fruit Ca content by prioritizing fertilization during the early stages of fruit development.

## 1 INTRODUCTION

Fruit quality is of utmost importance and interest in the cranberry (*Vaccinium macrocarpon* Ait.) industry due to its pivotal role in the production of higher‐value products, such as sweetened dried cranberries (SDCs) (Gallardo et al., 2018). The quality of SDC production and its efficiency have been linked to fruit firmness and internal fruit structure (Diaz‐Garcia et al., 2019; Lopez‐Moreno et al., 2023, 2024). These fruit quality traits are critical for the intensive SDC processing in which fruit is slowly frozen for storage, then thawed, sliced, and infused with a high‐temperature sugar solution for several hours (Grace et al., 2012). Fruit Ca plays an important role by forming insoluble pectin that strengthens the cell‐to‐cell adhesion in the middle lamella, thus positively affecting fruit firmness and pulp integrity in blueberry (Lin et al., 2019; Montecchiarini et al., 2021) and apple fruit (Ng et al., 2013).

Ca is translocated through the xylem into the fruit (Himelrick and McDuffie, 1983; Hocking et al., 2016; Montanaro et al., 2010), and its transport is driven by the water potential differential generated by fruit transpiration (Himelrick and McDuffie, 1983; Miqueloto et al., 2014; Montanaro et al., 2015). In cranberry fruit, stomata are only located inside the calyx lobes at the apex of the ovary (Vander Kloet and Others, 1988; Workmaster et al., 1999). Compared to leaves, fruits transpire less due to a thicker cuticle and lower stomata density (Martin and Rose, 2014). This condition has been found to limit Ca translocation into the apple fruit, leading to Ca‐deficient physiological disorders like bitter pit (Griffith and Einhorn, 2023). Fruit transpiration declines through fruit development due to physiological and anatomical changes in the epidermis and pedicel of the fruit (Mazzeo et al., 2013; Montanaro et al., 2015; Winkler et al., 2021). The primary changes in the epidermis during fruit development include a decrease in stomata density due to fruit expansion (Blanke and Leyhe, 1987; Peschel et al., 2003) and reduced stomatal functionality caused by stomatal occlusion from cuticle accumulation, as described in sweet cherry (*Prunus aviu*m L.;Knoche et al., 2001; Peschel et al., 2003), kiwifruit (*Actinidia deliciosa*; Morandi et al., 2012), strawberry (*Fragaria x ananassa* Duch; Winkler et al., 2021), mango (*Mangifera indica* L.; Higuchi and Sakuratani, 2006), and grape (*Vitis vinifera* L.; Palliotti and Cartechini, 2001). The fruit cuticle, which serves as a physical barrier against abiotic and biotic stress, regulates epidermal permeability to water loss through changes in its composition and thickness during fruit development (Erndwein et al., 2023; Lara et al., 2004; Riederer and Schreiber, 2001; VanderWeide et al., 2022). Increase in the cuticle thickness has been associated with declining rates of fruit transpiration in wine grapes (Becker and Knoche, 2012; Rogiers et al., 2004) and kiwifruits (Celano et al., 2009).

Declines in fruit transpiration and Ca accumulation in fruits are also influenced by reduction in water flow into the fruit, which results from a loss in pedicel xylem functionality (Bondada et al., 2005; Choat et al., 2009; Greenspan et al., 1994; Keller et al., 2006; Rogiers et al., 2004; Song et al., 2018). This loss of xylem functionality may be caused by either blockages or embolisms in the xylem vessels (Knipfer et al., 2015; Nordey et al., 2015) or by anatomical changes in the xylem vessels themselves (Dražeta et al., 2004). These changes in the pedicel xylem include reduced vessel diameter and increased vessel density, likely due to the expansion of the pith and phloem, which compresses and compromises xylem integrity (Jing et al., 2023; Lang and Ryan, 1994; Miqueloto et al., 2014; Rančić et al., 2010). These changes in the pedicel xylem are more likely to occur in the distal portion of the pedicel, where smaller vessel sizes create a “bottleneck” effect, increasing water resistance and interrupting water flow (Dražeta et al., 2004; Mazzeo et al., 2013; Song et al., 2018). In fact, exogenous applications of plant growth regulators that delay changes in the xylem pedicel have been shown to extend the period of water and Ca translocation into the fruit, highlighting the critical role of the pedicel in Ca accumulation (Griffith and Einhorn, 2023). In cranberry, little is known about Ca accumulation and how and why it varies over time to impact final fruit quality at harvest. Therefore, the objective of this study was to evaluate cranberry fruit transpiration, cuticle thickness, and pedicel vessels during cranberry fruit development, and to assess their impact on Ca translocation into the fruit. The knowledge generated in this research will help growers produce better quality fruit for SDC processing by managing nutrient application during fruit development.

## 2 MATERIALS AND METHODS

### 2.1 Site description and plant material

This study was conducted in the field at the Wisconsin Cranberry Research Station in 2022, near Black River Falls, WI (lat. 44°11′08.5“ N long. 90°44′18.7” W), using the cranberry (*Vaccinium macrocarpon* Ait) cultivar ‘Stevens'. Nutrient levels from cranberry leaf tissue analysis were within adequate levels for fruit production (Table S1). Soil analysis from the site showed a typical cranberry farm profile with 0.2% organic matter and pH 5.7 (Table S2). Standard commercial and cultural practices for cranberry production were implemented at the research station (Sandler and DeMoranville, 2009), including fertilization, pest and insect management, watering, winter protection, and sanding of beds (rejuvenation) every three years for sanitation and rooting of new growth. The maximum, minimum, and average ambient temperatures were recorded hourly using a shielded Hobo pendant data logger (Onset Computer) placed at canopy level. Growing degree days (GDD) were calculated using a modified version of the equation used by DeVetter et al. (2013), incorporating a base temperature of 5°C (Table 1). The summation of GDD began on June 2 until the last collection date. The 30‐year average annual maximum and minimum temperatures, and precipitation at Black River Falls were 12.5°C, 0.72°C, and 838 mm, respectively (NOAA MRCC, 2024).

**TABLE 1:** Cranberry growing average maximum and minimum temperatures (°C), and GDD from July to August in 2022 near Black River Falls in Central Wisconsin, USA.

Month	Average max temperature	Average min temperature	GDD
July	31.91	10.82	168
August	30.17	10.8	318
September	27.31	11.53	433

### 2.2 Fruit growth and development

Fruit growth and development were characterized by recording the average individual berry fresh and dry weight, and size using using a total of five replicates of 40 g of berries collected from the two basal positions in the upright (i.e., vertical stems containing a terminal apical bud). Sampling began seven days after full bloom (DAFB) and continued until two weeks before harvest, for a total of 10 sample collections during the growing season.

### 2.3 Fruit Ca content

For each sample collection date, a total of five replicates of 40 g of berries were collected from the two basal positions in the upright, and samples were oven‐dried at 70°C until constant weight. The samples were ground using a mortar and pestle and then stored in 20 mL scintillation vials. Ca concentration was measured using a 100 mg aliquot of ground sample digested using nitric acid in hydrogen peroxide solution and analyzed via ICP‐OES at the Soil and Forage Analysis Laboratory of the University of Wisconsin‐Madison. The Ca concentration was expressed as ppm (mg kg^−1^ DW) and converted to fruit Ca content in mg per berry.

### 2.4 Fruit transpiration rate

Fruit transpiration was measured using a CIRAS‐3 gas exchange system (PP Systems, Amesbury MA) configured with a conifer chamber. In the field, ten randomly selected fruits attached to the most basipetal position on the upright were measured on each collection date. The two leaf‐like bracts that occur along the fruit pedicel were removed from the conifer chamber due to possible interference with fruit transpiration. Measurements were taken in the morning with a cuvette flow of 400 cc min^−1^, analyzer flow of 100 cc min^−1^, ambient light source, cuvette temperature of 27°C, boundary layer resistance of 0.40 m^−2^ s^−1^ mol^−1^, and stomatal ratio of 10%. Fruit transpiration rate (E) was expressed in mmol m^−2^ s^−1^. In addition, to assess the relative contributions of the fruit cuticle and stomata to transpiration, fruit transpiration was measured in ‘Stevens’ plants grown at a UW‐Madison campus greenhouse at 25°C, 80% RH, long day light conditions (16 h day, 8 h night), and manually pollinated as part of the cranberry breeding program at UW‐Madison. Fruit stomata present in the calyx were covered with vacuum grease prior to measuring fruit transpiration in the greenhouse. Due to the low success rate of hand‐pollination in cranberry, only four fruits from a single plant were selected, and transpiration of each fruit was measured before and after blocking the stomata. Based on fruit size and weight, the phenological stage of the greenhouse fruit corresponded to fruit in field conditions at 7 DAFB.

### 2.5 Stomata density

**FIGURE 1:**
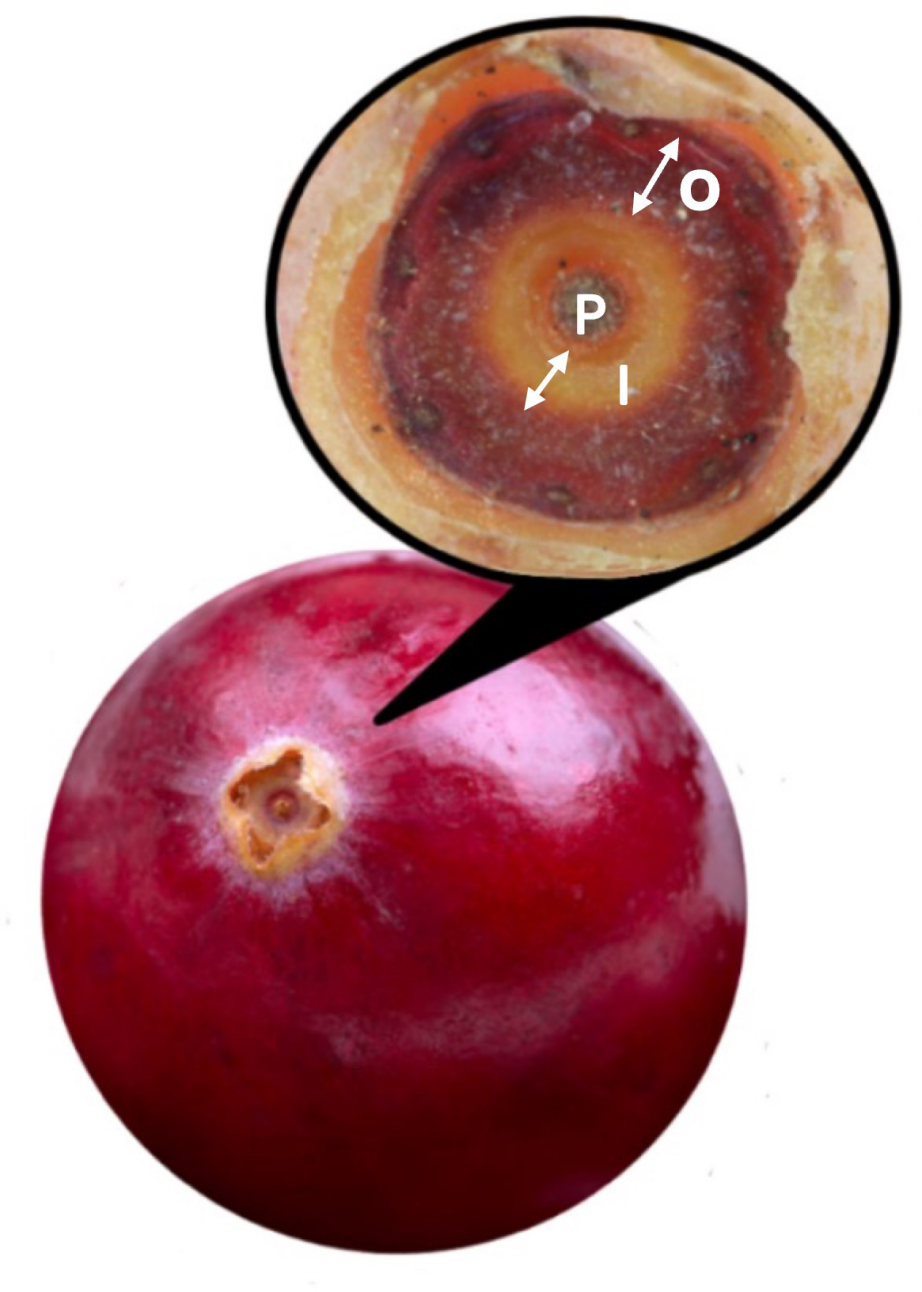
Illustration of the section inside the calyx‐bounded area of the cranberry fruit, showing the position of the pistil scar (P), the inner ring (I) and the outer ring (O). Stomata are only located in the outer ring.

For each field collection date, a total of five fruits from the most basipetal position in the upright were randomly selected. Unlike blueberry fruits where stomata are present on different fruit sections (Yang et al., 2020), cranberry fruit stomata are only located inside the calyx lobes at the apex of the ovary (Vander Kloet and Others, 1988; Workmaster et al., 1999). To measure stomata density, only the area inside the calyx lobes at the apex of the ovary was coated with clear lacquer (nail polish) and removed once dried. The dried lacquer was then placed on a glass slide for microscopy observation at 10× magnification using an Olympus BX50 (Olympus Optical Company) and images were captured with a Canon digital camera (EOS Rebel T6i). For each image, the radius of the calyx‐bounded area was recorded using ImageJ software version 1.X (Schneider et al., 2012) and the area was divided into two sections: an outer ring containing stomata and an inner ring without stomata (Figure 1). For each section, the total number of stomata was counted from a randomly selected 0.4 × 0.4 mm area in the outer ring. The stomata density per section was calculated by dividing the total number of stomata by the area. The total number of stomata per fruit was calculated by multiplying the stomata density by the area of the inner calyx‐bounded region. In addition, stomata from fresh fruit samples were observed using an environmental scanning microscope (ESEM) to assess the amount of wax covering them. Images were captured at 10 kV with an SEM (S‐570 LaB_6_; Hitachi) at 750× magnification. Five images, each representing a different fruit, were taken on each collection date.

### 2.6 Fruit cuticle thickness sample preparation and analysis

A total of five independent fruits were randomly collected from the field on each collection date, starting 14 DAFB and continuing until 84 DAFB. Transversal fruit sections were prepared for light microscopy by fixing the samples overnight at 4°C in a solution of 4% (w/v) glutaraldehyde (Sigma–Aldrich) and 0.5 mol L^−1^ potassium phosphate buffer (pH 7.5) (Bolivar‐Medina et al., 2019). The samples were then embedded in medium‐grade LR white resin (Ted Pella, Inc.) by gradually replacing ethanol with LR white. A series of eight thin transverse sections (2 μm) were cut from two replicates per collection date using an ultramicrotome (MT‐2; Sorvall). All observations were made using a bright‐field microscope (Olympus BX50 at 40×). Images were captured from two of the eight sections per replicate using a Canon digital camera (EOS Rebel T6i). Cuticle thickness was estimated by randomly measuring the width (μm) of the cuticle at 25 different points around the epidermis in each sectioning the software ImageJ, totaling 100 measurements per collection date. Additionally, fruit cuticle thickness was also measured from the fruit in greenhouse conditions using the same methodology.

### 2.7 Pedicel and fruit xylem functionality using dye tracing assay

**FIGURE 2:**
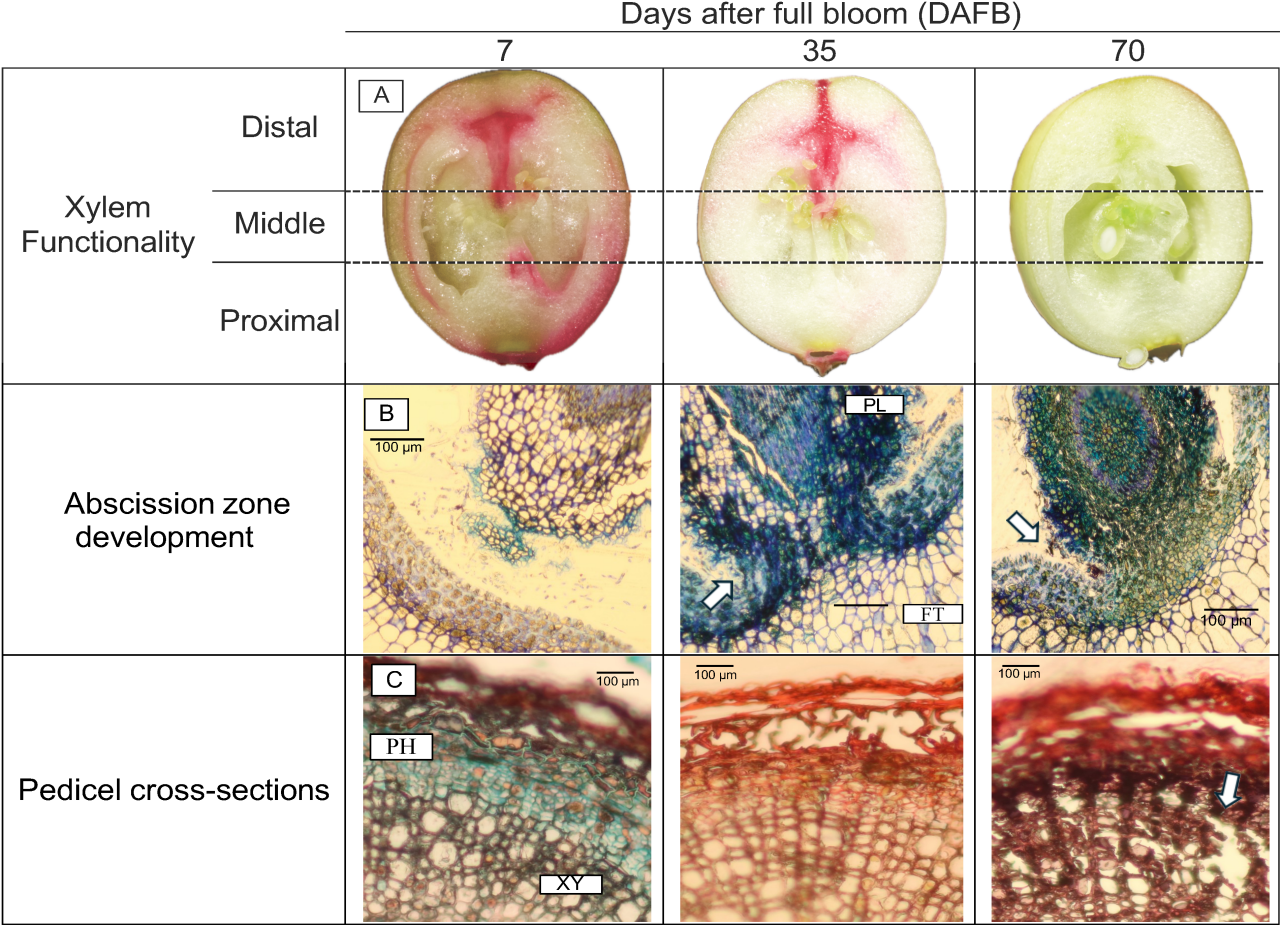
Cranberry fruit acid fuchsin trace (A), pedicel abscission zone (B), and pedicel cross‐section (C) of xylem (XY) and phloem (PH) vascular bundles compared between 7, 35, and 70 DAFB in ‘Stevens’ grown near Black River Falls in Central Wisconsin, USA. In A, the presence or absence of acid fuchsin was evaluated in three different fruit portions (distal, middle, and proximal). In B, the arrow indicates the point of attachment between the fruit (FT) and the pedicel (PL), where there is a progression in the tissue rupture between 35 and 70 DAFB marked by the arrows. In C, the arrow at 70 DAFB indicates xylem vessel rupture.

Pedicel and fruit xylem functionality was assessed using a 1% (w/v) acid fuchsin staining solution, following the methodology described by Dražeta et al. (2004) with some modifications. For each collection date, a total of 20 uprights containing fruit were randomly collected and divided into two separate assays. In the first assay, 10 upright stem bases were cut under water to prevent cavitation and then submerged in acid fuchsin for 30 minutes. In the second assay, 10 fruits with their pedicels were detached from 10 independent uprights; the proximal portion (closer to the upright stem) of each pedicel was submerged in acid fuchsin for 30 minutes. Both assays were conducted inside a laminar fume hood at room temperature to ensure consistent airflow. After 30 minutes in the first assay, two fruits from the most basipetal position on the upright were detached and longitudinally cut with a razor blade. For the second assay, the same procedure was done for detached fruits. One cut surface was observed using a dissecting microscope (Olympus SZX12; Tokyo, Japan) at 1× magnification. Images were captured using a Canon digital camera. The fruit longitudinal cut was then divided again into three transverse sections, and each section was visually evaluated for the presence or absence of the dye (Figure 2A). Additionally, a digital automated method was developed to measure the percentage of dye coverage on the total cut surface area, complementing the visual evaluations. First, the background was removed using the ‘rembg’ and ‘PIL’ packages in Python programming language Python Software Foundation, https://www.python.org/). The percentage of red color was calculated by subtracting the red pixels from the rest of the fruit area. Customized code is available in GitHub repository (rojasbarros/dye_assay).

### 2.8 Fruit pedicel microscopy sample preparation and vascular tissue analysis

For all collection dates, three fruit pedicels were randomly selected and fixed following the methodology described for evaluating fruit cuticle thickness. Each pedicel was then divided into three equal portions: the proximal (closer to the upright stem), the middle (between proximal and distal ends), and the distal portion (closer to the fruit). The proximal portion was also used to describe the development of the fruit abscission zone. A series of eight thin transverse sections (2 μm) from three pedicels at each collection date were cut using an ultramicrotome for vascular tissue analysis. The same methodology was used for the description of the abscission zone, but using thin longitudinal sections from samples collected only at 7, 35 and 70 DAFB.

Following the protocol described by Song et al. (2018), transverse sections of the pedicel were successively stained with 1% (w/v) safranin O (Sigma–Aldrich) and Fast Green FCF at a concentration of 0.5 g in 100 mL of 95% ethanol (Sigma–Aldrich). Longitudinal sections were stained with 0.05% (w/v) Toluidine Blue O (Sigma‐Aldrich). Each pedicel section was examined on an Olympus BX50 at 40× magnification to measure xylem vessel diameter and vascular tissue diameter, as well as at 20× magnification for additional vascular tissue diameter measurements and abscission zone characterization. Four of the eight sections were photographed using a Canon digital camera, resulting in a total of 360 images across the 10 collection dates for subsequent analysis of xylem vessel development and vascular tissue diameter.

From each image, three independent areas of 1000 μm^2^ on the xylem portion of the pedicel, previously identified with safranin O, were randomly selected using ImageJ. The selected areas were analyzed using the ‘Analyze Particle’ function to estimate the individual vessel area, perimeter, diameter, total vessel number, vessel density, and hydraulic weighted mean diameter (Dh) using the equation (1) described (Kolb and Sperry, 1999):

(1) $$\mathrm{Dh}=\frac{\sum d^{5}}{\sum d^{4}}$$

In addition, five independent measurements from the xylem, phloem, and pith radius (μm), were performed to estimate the change in proportion of the vascular tissue through fruit development.

### 2.9 Statistical analyses

Fruit Ca accumulation over time was analyzed using a quadratic regression following the equation:

(2) $$\log\left(\hat{Y}i\right)={\hat{\beta }}_{0}+{\hat{\beta }}_{1}\left(x_{1i}\right)+{\hat{\beta }}_{2}\left({x^{2}}_{1i}\right)+\varepsilon$$

in which $\hat{Y}i$ is the fruit Ca content, ${\hat{\beta }}_{0}$ is the intercept, $x_{1}$ is DAFB, $x_{2}$ is DAFB^2^, and ε the error. The highest fruit Ca content accumulation was calculated using equations 3 and 4:

(3) $$\log\left(y\right)=\left(\beta_{0}\right)+\left(\beta_{x1}\right)x-\left(\beta_{x2}\right)x^{2}$$

(4) $$\frac{\;{\hat{\beta }}_{1}}{2{\hat{(\beta }}_{2})}$$

A multiple linear regression (MLR) was used to analyze the relationship between fruit Ca content and all the other physiological and anatomical parameters measured. An exhaustive model search, considering an adjusted r^2^, Akaike's information criterion (AIC), and Schwarz's Bayesian information criterion (BIC), was used to identify the best model explaining the response of fruit Ca content. The best model considered only one parameter described in the following equation:

(5) $$\left(\hat{Y}i\right)={\hat{\beta }}_{0}+{\hat{\beta }}_{1}\left(x_{1i}\right)+\varepsilon$$

in which $\hat{Y}i$ is the fruit Ca content, ${\hat{\beta }}_{0}$ is the intercept, $x_{1}$ is fruit transpiration (E), and ε the error. Similarly, an exhaustive model search was used to identify the best model explaining the response of fruit transpiration. The best model considered three parameters described in the following equation:

(6) $$\hat{Y}i={\hat{\beta }}_{0}+{\hat{\beta }}_{1}x_{1i}+{\hat{\beta }}_{2}x_{2i}+{\hat{\beta }}_{3}x_{3i}+\varepsilon$$

in which $\hat{Y}i$ is the fruit transpiration (E), ${\hat{\beta }}_{0}$ is the intercept, $x_{1}$ is Dh (1), $x_{2}$ is xylem vessel density, $x_{3}$ is cuticle thickness, and ε the error.

Xylem functionality was analyzed using a logistic regression to calculate the probability of the presence (one) or absence (zero) of acid fuchsin dye in each of the three fruit sections across collection dates, resulting in the following model (4):

(7) $$P\left(\mathrm{Yi}\right)=\ln\left(\frac{\pi }{1-\pi }\right)={\hat{\beta }}_{0}+{\hat{\beta }}_{1}x_{1i}+{\hat{\beta }}_{2}x_{2i}+\varepsilon$$

in which Yi is the probability of presence or absence of the dye in the fruit portion, ${\hat{\beta }}_{0}$ is log odds of the dye when $x_{1}$ and $x_{2}$ are equal to zero, $x_{1}$ is DAFB, $x_{2}$ is the fruit section (proximal, middle, and distal), and ε the error.

An analysis of variance (ANOVA) was performed to test the changes in xylem vessel diameter, density, Dh, and vascular tissue diameter across the different fruit sections and collection dates. For each parameter, a pairwise comparison was performed using the estimated marginal means (EMMs) and Tukey's HSD range test with an α‐level of 0.05. The assumptions of normality, linearity, and constant variance were assessed in every analysis, except for the logistic regression, by using residual vs. fitted plots, studentized Breusch‐Pagan test, and Shapiro–Wilk normality test. The variance inflation factor (VIF) was used in the MLR model for removing multicollinear parameters when the value was larger than 10. Assumption remedies were performed when needed, outliers were removed when Cook's distance value test was significant at α‐level of 0.05, and data outputs, and plots were generated using R Studio (R Core Team, 2023).

## 3 RESULTS

### 3.1 Fruit growth

**FIGURE 3:**
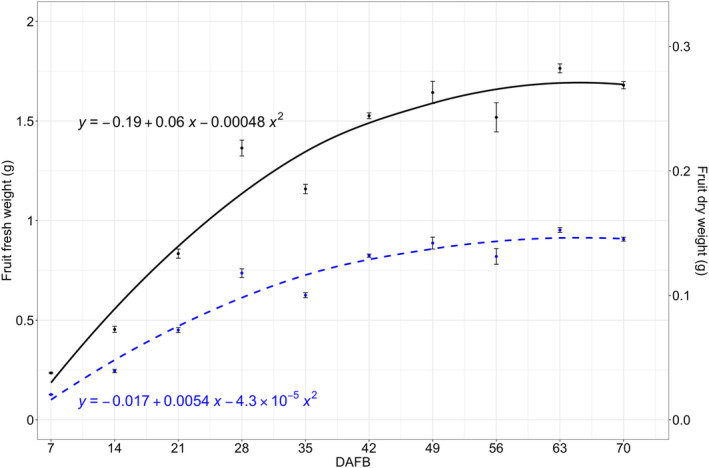
Cranberry fruit (*n* = 5 replicates, each being ca. 40 g of berries) fresh weight (g) (black line) and dry weight (blue dotted line) from 7 to 70 days after full bloom (DAFB) in ‘Stevens’ grown near Black River Falls in Central Wisconsin, USA.

Fruit growth exhibited a non‐linear pattern, with a rapid increase in fresh weight from 0.2 to 1.2 g per fruit during the first 35 DAFB, followed by a plateau at 1.7 g from 49 DAFB to 70 DAFB (Figure 3). Similarly, fruit dry weight showed a similar pattern to fresh weight; however, the accumulation of dry matter was significantly slower, as indicated by the differences in the slopes of the quadratic equations in Figure 3. The GGD accumulation followed a linear pattern, increasing from 100 to 400 GDD throughout the growing season (Table 1).

### 3.2 Fruit Ca content

**FIGURE 4:**
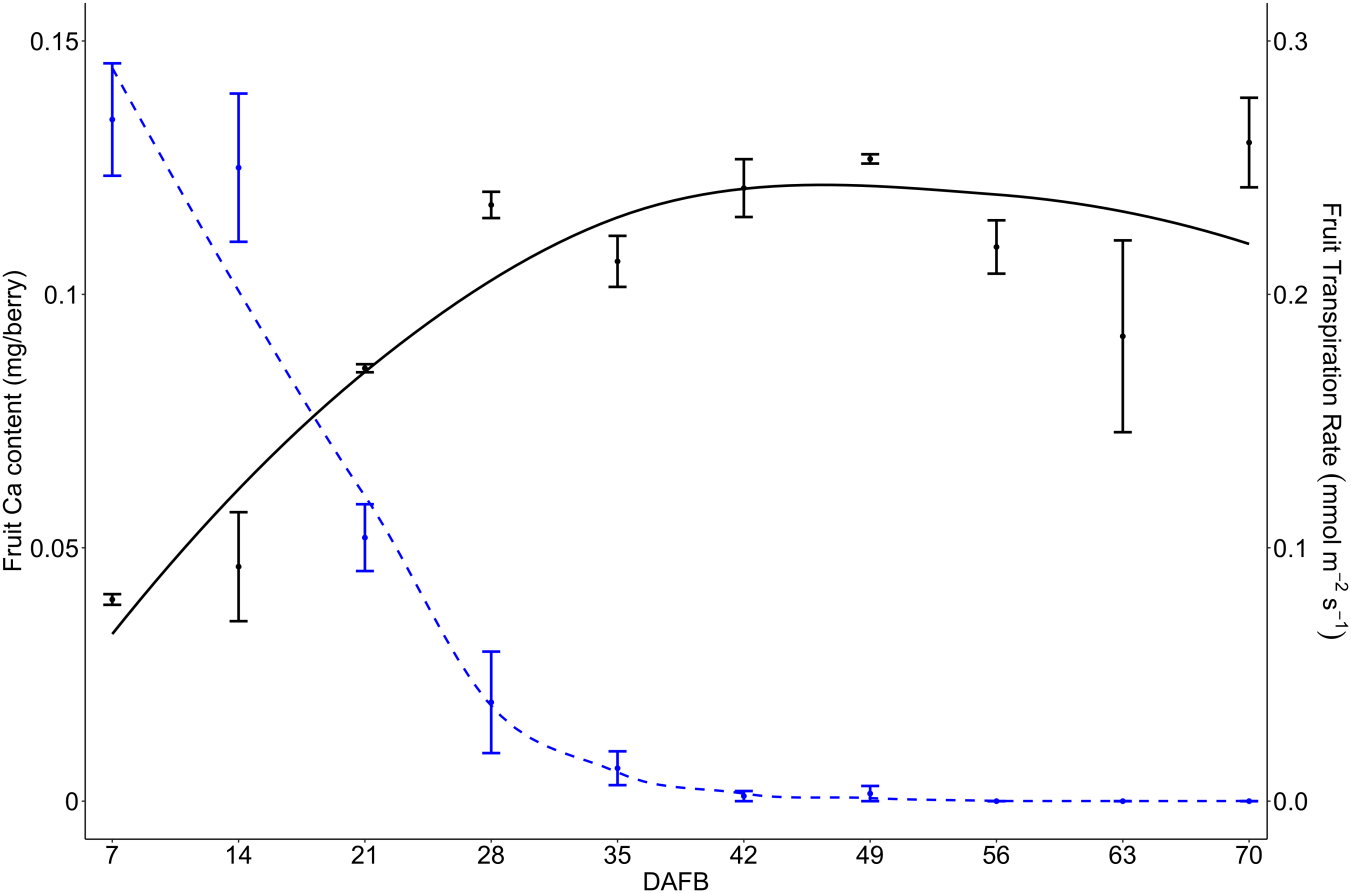
Cranberry fruit (n = 5 replicates, each being ca. 40 g of berries) calcium (Ca) content (mg) of berry dry weight (black line) in relationship with fruit transpiration (mmol/m^2^/s^−1^, *n* = 40) (dashed blue line) from 7 to 70 days after full bloom (DAFB) in ‘Stevens’ grown near Black River Falls in Central Wisconsin, USA.

The fruit Ca content exhibited a non‐linear pattern, rapidly increasing from 0.04 to 0.1 mg per berry within the first 21 DAFB. It reached maximum Ca accumulation of 0.14 mg per berry at approximately 42 DAFB (see equations 3 and 4 with data from Table 2) and remained constant until 70 DAFB (Figure 4).

**TABLE 2:** Linear regression output of the relationship between cranberry fruit calcium (Ca) content in mg per berry and days after full bloom (DAFB) in ‘Stevens’ grown near Black River Falls in Central Wisconsin, USA.

Coefficients	Estimates	SE	t‐value	p‐value	r^2^	Adj. r^2^
Intercept ($\beta_{0}$)	−3.5	0.077	−45.51	< 2e^‐16*^	0.864	0.858
DAFB ($\beta_{x1}$)	0.39	0.032	12.39	4.25 e^‐16*^		
DAFB^2^ ($\beta_{x2^{2}})$	−0.026	0.002	−9.42	3.28 e^‐12*^		

* The p‐value <0.05 are significant different at α = 0.05.

### 3.3 Fruit transpiration rate

The average field fruit transpiration rate exhibited a decreasing non‐linear pattern, declining from 0.13 to nearly 0 mmol m^−2^ s^−1^ within the first 35 DAFB (Figure 4). Fruit from the greenhouse study, which corresponded to fruit in field conditions at 7 DAFB, was used to compare the contribution of the fruit cuticle versus stomata to transpiration. The fruit recorded an average fruit transpiration before covering the fruit stomata with vacuum grease of 0.33 (± 0.03) mmol m^2^ s^−1^. After covering the stomata with vacuum grease, the fruit transpiration decreased to 0.29 (± 0.01) mmol m^−2^ s^−1^ (Table 3).

**TABLE 3:** Cranberry fruit cuticle thickness (μm), size (mm^2^), and transpiration rate (mmol m^−2^ s^−1^) before and after covering all fruit stomata with vacuum grease in ‘Stevens’ grown under greenhouse conditions in WI.

Fruit number	Cuticle thickness (μm)	Fruit size (mm^2^)	Fruit transpiration (mmol m^−2^ s^−1^)	Fruit transpiration with covered stomata (mmol m^−2^ s^−1^)	Difference in transpiration (%)
1	3.74	66	0.36	0.29	−0.19
2	4.08	112	0.31	0.31	0.00
3	5.14	72	0.34	0.29	−0.15
4	4.01	96	0.30	0.28	−0.07

### 3.4 Stomatal density

The stomata density per μm^2^ and the total stomata number per fruit remained unchanged through fruit development (Table 4).

**TABLE 4:** Cranberry fruit average stomata density per area (mm^−2^) and number of stomata per fruit from 7 to 70 days after full bloom (DAFB) in ‘Stevens’ grown near Black River Falls in Central Wisconsin, USA.

DAFB	Stomata density per area (mm^−2^)	Stomata number per fruit
7	147	321
14	134	317
21	115	322
28	119	311
35	131	314
42	152	303
49	145	317
56	135	330
63	127	315
70	141	318
p‐value	0.065	0.098

Means presented with no letters within a table column are not different at *p* < 0.05 using Tukey's HSD comparison.

### 3.5 Cuticle thickness

**FIGURE 5:**
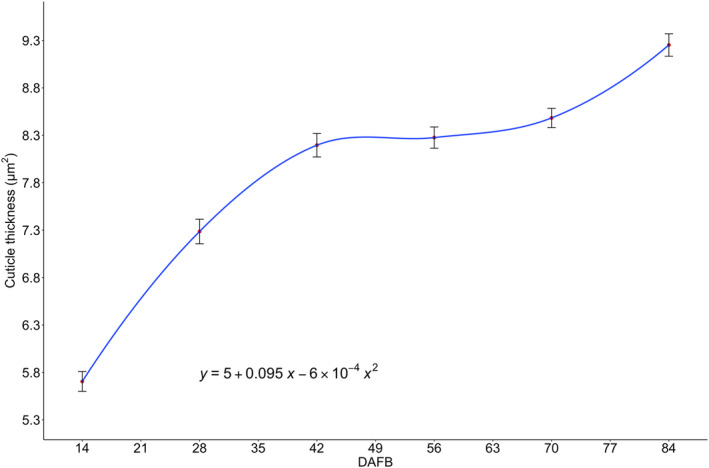
Cranberry fruit cuticle thickness (μm) of fruit (n = 5) collected from 14 to 84 days after full bloom (DAFB) in ‘Stevens’ grown near Black River Falls in Central Wisconsin, USA.

Cuticle thickness followed a double‐sigmoidal pattern explained by the quadratic equation in Figure 5, which was characterized by a quick increase in thickness from 5.6 to 8.2 μm during the first 42 DAFB, followed by a plateau where no differences were observed between 42 and 56 DAFB, until another slight increase in thickness occurred from 56 to 84 DAFB with a maximum of 9.3 μm. Fruit cuticle thickness under greenhouse conditions, which corresponded to fruit in field conditions at 7 DAFB, average 4.42 μm (± 0.6) (Table 3).

### 3.6 Xylem vessel functionality

**FIGURE 6:**
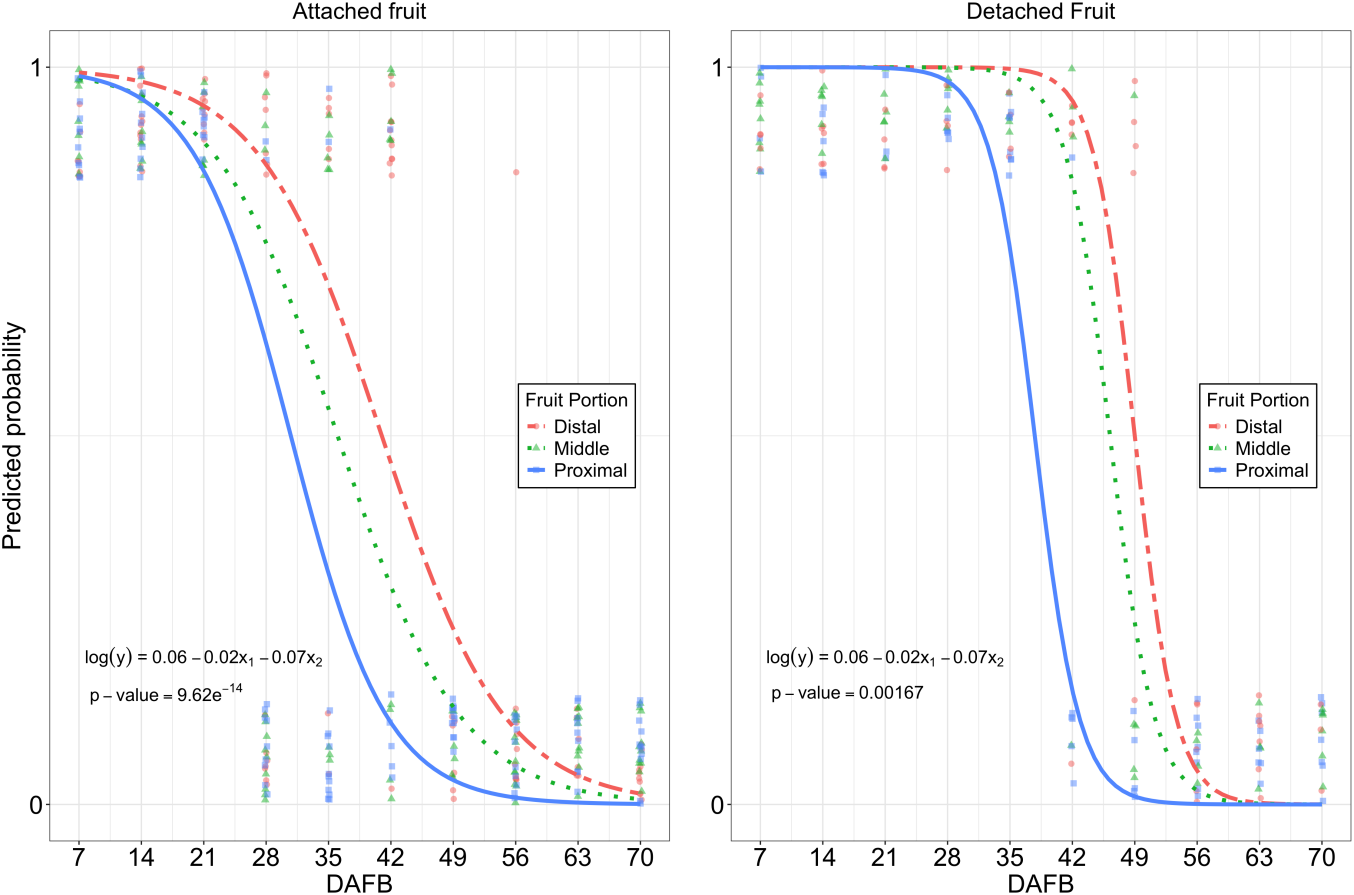
Comparison of the predictive probability of acid fuchsin presence or absence in each of the three equally divided transverse sections (proximal portion closer to the pedicel, middle between proximal and distal, and distal portion closer to the calix of the fruit) of attached (*n* = 10) and detached (n = 10) ‘Stevens’ cranberry fruit collected from 7 to 70 days after full bloom (DAFB) near Black River Falls in Central Wisconsin, USA.

**FIGURE 7:**
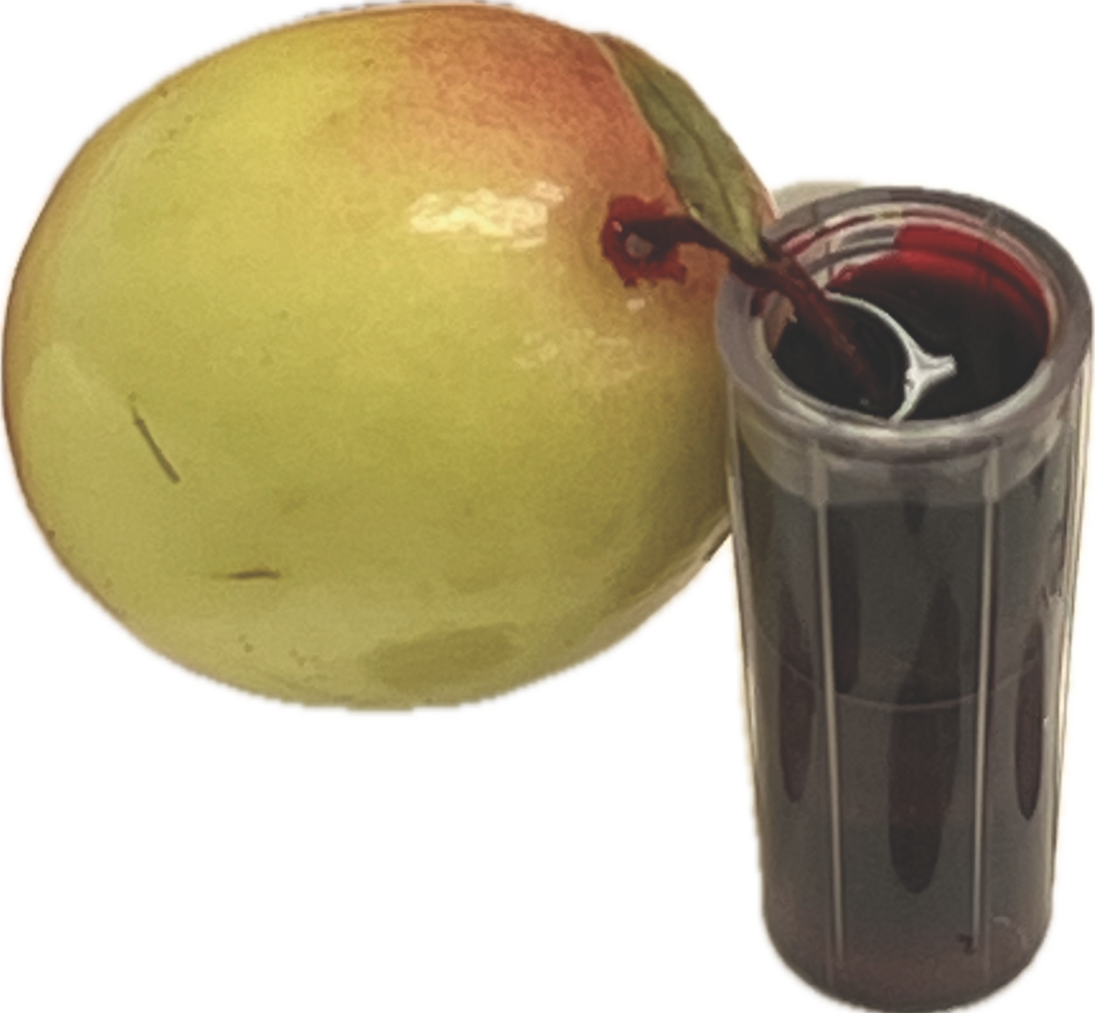
Detached fruit from a cranberry upright showing dye leakage at the pedicel receptacle during a dye trace assay using a 1% (w/v) acid fuchsin staining solution in cranberry ‘Stevens’ at 70 days after full bloom (DAFB) grown near Black River Falls in Central Wisconsin, USA.

A logistic regression was used to predict the probability of finding dye in the different portions of the fruit under two assay conditions: fruit attached and detached from the upright stem. The overall probability of detecting dye in any portion of the attached fruit decreased between 28 and 49 DAFB, after which the probability approached nearly zero at 56 DAFB (Figure 6). Within the fruit, the overall probabilities of detecting dye in each fruit portion, with DAFB held constant, were 0.59, 0.41, and 0.19 for the distal, middle, and proximal portions, respectively (Table 5). Dye was visible in all three portions of the fruit at 28 DAFB, but its presence began to decline, with the proximal portion showing the first absence of dye at 35 DAFB. This decline continued in the middle and distal portions until 70 DAFB. Automated image analysis of the total fruit area covered with dye revealed a similar pattern to the visual evaluations, showing a clear decrease in dye presence after 49 DAFB (Table 6). The detached fruit exhibited a comparable pattern to the attached fruit, but the overall probability of dye detection in each fruit portion was higher during the same period compared to the attached fruit, with values of 0.99, 0.96, and 0.43 in the distal, middle, and proximal portions, respectively (Table 5). The automated image analysis also aligned with the visual evaluations, demonstrating a significant decline in presence after 49 DAFB (Table 6). Additionally, a few detached fruits showed dye leakage at the pedicel receptacle (i.e., the area where the flower organ grows), starting at 49 DAFB, and by 70 DAFB, all evaluated fruits exhibited this condition (Figure 7), possibly due to the development of the pedicel abscission zone (Figure 2B).

**TABLE 5:** Comparison between attached and detached cranberry fruit on the estimated predicted probability of presence or absence of 1% acid fuchsin (w/v) in each of the three equally divided transverse sections (proximal portion closer to the pedicel, middle between proximal and distal, and distal portion closer to the calix of the fruit) of ‘Stevens’ fruit across all 10 collection dates grown near Black River Falls in Central Wisconsin, USA.

	Fruit portion	Attached	Detached
Estimated Predicted Probability	Proximal	0.59	0.99
	Middle	0.41	0.96
	Distal	0.19	0.43

**TABLE 6:** Estimation of chroma, luminance, hue (°), and dye presence (%) of cranberry fruit with 1% acid fuchsin (w/v) in attached and detached longitudinal ‘Stevens’ fruit cuts from 7 to 70 days after full bloom (DAFB) grown near Black River Falls in Central Wisconsin, USA.

DAFB	Type of fruit	Chroma	Luminance	Hue (°)	Dye presence (%)
7	Attached	0.06	0.13	6.67	42.0
	Detached	0.04	0.09	7.06	26.3
14	Attached	0.01	0.02	13.7	10.1
	detached	0.03	0.04	11.2	27.9
21	Attached	0.01	0.01	14.4	6.3
	Detached	0.02	0.03	9.71	24.5
28	Attached	0.02	0.04	10.5	11.5
	Detached	0.03	0.07	8.1	24.4
35	Attached	0.01	0.03	11.5	6.4
	Detached	0.02	0.04	3.6	9.7
42	Attached	0.01	0.03	10.9	7.6
	Detached	0.01	0.02	8.76	4.6
49	Attached	0.00	0.00	−5.16	0.5
	Detached	0.00	0.00	−6.03	0.5
56	Attached	0.00	0.00	−3.4	0.3
	Detached	0.00	0.00	−2.85	0.2
63	Attached	0.00	0.00	0.00	0.0
	Detached	0.00	0.00	0.00	0.0
70	Attached	0.00	0.00	0.00	0.0
	Detached	0.00	0.00	0.00	0.0

### 3.7 Pedicel vascular bundle anatomy

**FIGURE 8:**
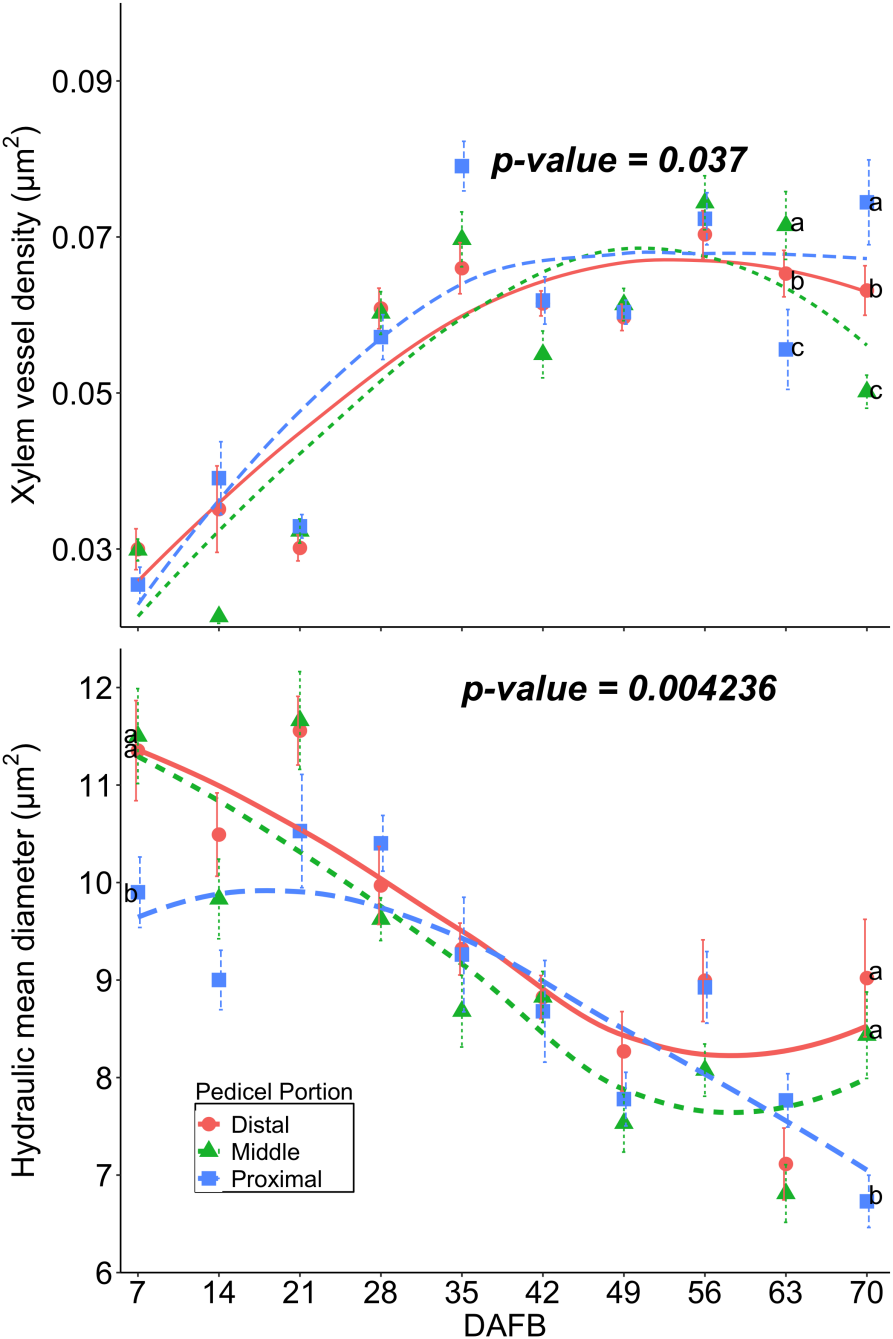
Cranberry xylem vessel average density (μm^2^) and hydraulically weighted mean diameter (Dh) in (μm) of different pedicel portions (*n* = 3): proximal (closer to the upright stem), the middle (between proximal and distal ends), and the distal portion (closer to the fruit), collected from 7 to 70 days after full bloom (DAFB) in ‘Stevens’ grown near Black River Falls in Central Wisconsin, USA.

The density of the pedicel xylem vessels exhibited a non‐linear pattern, showing a rapid increase from 0.03 to 0.07 μm^2^ during the first 35 DAFB, followed by no further changes until the end of the growing season (Table 7). When evaluating the xylem vessel density for each portion of the pedicel separately, differences were observed between 63 and 70 DAFB (Figure 8). Notably, the xylem vessel density in the proximal portion of the pedicel (i.e., closer to the abscission zone) was higher compared to the other two portions.

**TABLE 7:** The average xylem vessel density (μm^2^) and hydraulically weighted mean diameter (Dh) in μm from pedicels collected from 7 to 70 days after full bloom (DAFB) in ‘Stevens’ grown near Black River Falls in Central Wisconsin, USA.

	Pedicel xylem vessels	
DAFB	Density per area (μm^2^)	Dh (μm)
7	0.03 a	11.2 ab
14	0.03 a	9.8 bc
21	0.03 a	11.4 a
28	0.06 bc	10.0 bd
35	0.07 bd	9.1 cde
42	0.06 c	8.8 cf
49	0.06 cd	7.9 gh
56	0.07 d	8.7 efg
63	0.06 cd	7.2 h
70	0.06 cd	8.1 fh
p‐value	<2e^‐16*^	<2e^‐16*^

Means presented with the same letter within a table column are not different at P < 0.05 using Tukey's HSD comparison.

The hydraulic weighted mean diameter (Dh), which represents the vessel diameter adjusted for the number of vessels, consistently decreased from 11.2 to 8.1 μm throughout fruit development (Table 7). In addition to the changes in the xylem vessel diameter and density, tissue rupture was observed in the xylem during the late stages of fruit development at 70 DAFB (Figure 2C). When analyzing the pedicel portions, the Dh of the proximal and middle portions was larger compared to the distal portion at both 7 DAFB and 70 DAFB (Figure 8).

**FIGURE 9:**
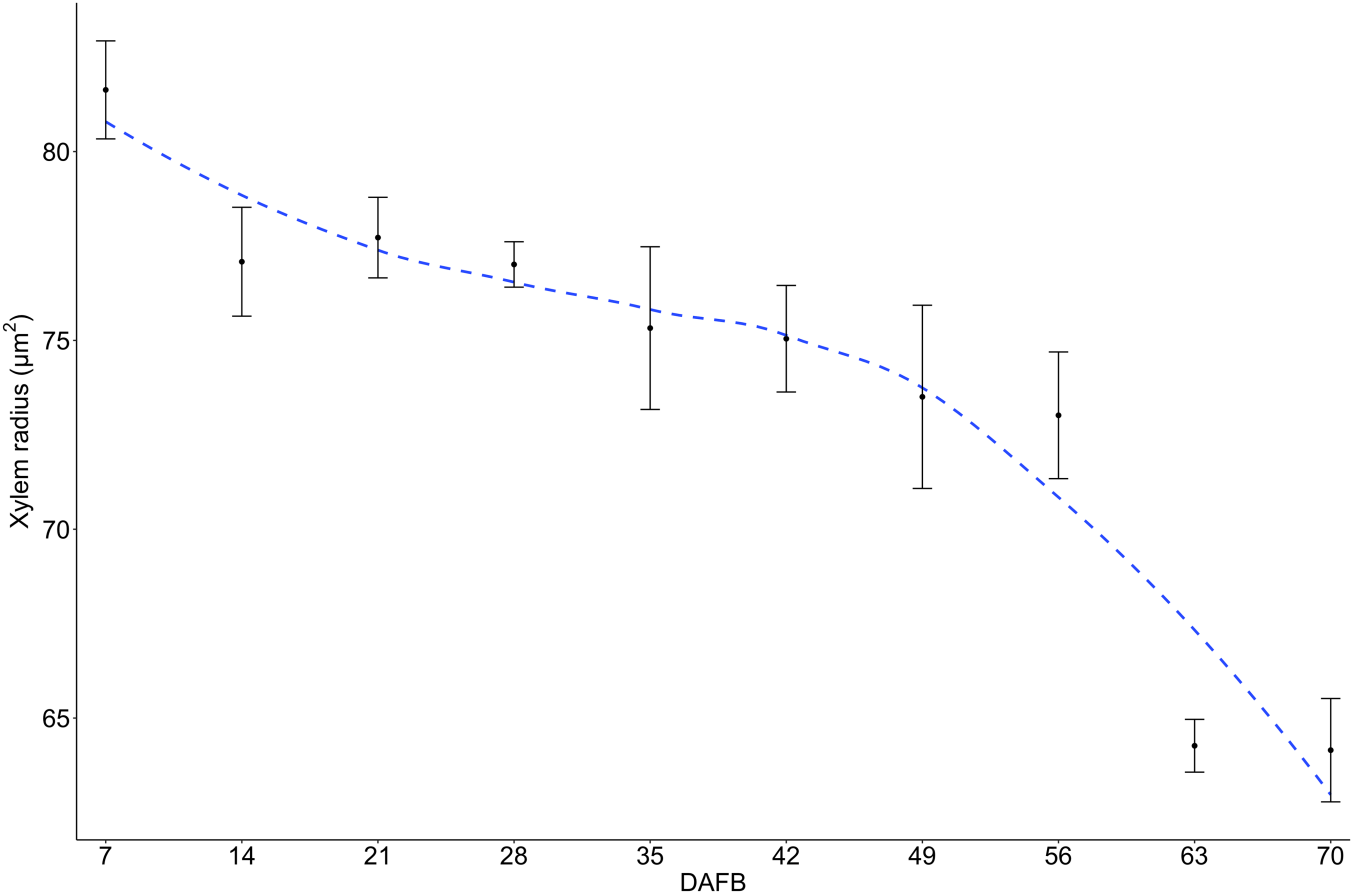
Cranberry xylem radius (μm) of fruit pedicels (n = 3) collected from 7 to 70 days after full bloom DAFB in ‘Stevens’ grown near Black River Falls in Central Wisconsin, USA.

**FIGURE 10:**
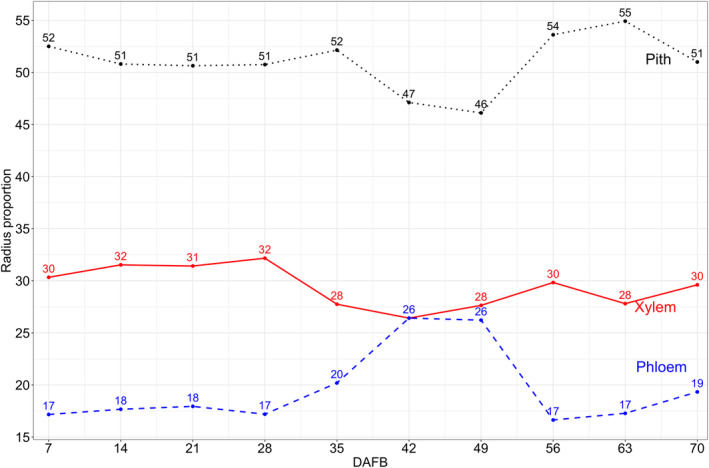
Cranberry diameter proportion (percentage) of pith (dotted line), xylem (straight line), and phloem (dashed line) of pedicels (n = 3) collected 7 to 70 DAFB in ‘Stevens’ grown near Black River Falls in Central Wisconsin, USA.

The xylem radius decreased from 83 to 64 μm between 7 and 70 DAFB (Figure 9). During this same period, the proportion of the pedicel phloem and pith radius relative to the xylem increased from 17 to 26% between 35 and 42 DAFB, and from 46 to 55% between 56 to 70 DAFB, respectively (Figure 10).

## 4 DISCUSSION

In this study, we evaluated the timing of Ca accumulation in developing ‘Stevens’ cranberry fruits and examined how this process was influenced by physiological and anatomical parameters that modulate fruit transpiration. Fruit Ca content increased during the first 28 DAFB and was regulated by fruit transpiration. Transpiration rates were affected by the increase in fruit cuticle thickness and anatomical changes in the pedicel xylem.

Ca content was strongly correlated with fruit transpiration (r^2^ = 0.92, Table 8), with the highest levels of fruit Ca content observed shortly after transpiration rates dropped to their lowest point (Figure 4). The exhaustive model selection indicated that transpiration was a better predictor of Ca accumulation than stomata conductance. This relationship between fruit Ca content and transpiration has also been reported in several many fruit crops, including northern highbush blueberry (*Vaccinium corymbosum*; Yang et al., 2020), kiwifruit (Montanaro et al., 2006; Morandi et al., 2012; Smith et al., 1995), apple (*Malus domestica* Borkh; Himelrick and McDuffie, 1983; Jones et al., 1983; Perring and Jackson, 1975; Wilkinson and Perring, 1964), apricot (*Prunus armeniaca* L.; Montanaro et al., 2010) mango (Higuchi and Sakuratani, 2006), and grape (Creasy et al., 1993).

**TABLE 8:** Linear regression output of the relationship between cranberry fruit calcium (Ca) content in mg per berry and fruit transpiration (mmol m^−2^ s^−1^) through fruit development in ‘Stevens’ grown near Black River Falls in Central Wisconsin, USA.

Coefficients	Estimates	SE	t‐value	p‐value	r^2^	Adj. r^2^
Intercept ($\beta_{0}$)	−2.14	0.047	−45.71	5.79e^‐11*^	0.93	0.92
Fruit transpiration ($\beta_{x1}$)	−3.81	0.385	−9.89	9.18e^‐06*^		

* The p value <0.05 are significant different at α = 0.05.

Changes in fruit transpiration rate during cranberry fruit development were not associated with changes in stomata density, as it has been described in other fruits such as northern highbush blueberry (Yang et al., 2020), peach (*Prunus persica L*. ‘nucipersica’; Gibert et al., 2010), kiwifruit (Montanaro et al., 2015; Morandi et al., 2012), strawberry (Winkler et al., 2021), and wine grape (Blanke and Leyhe, 1987). In cranberry fruit, stomata are located exclusively in the area within the calyx lobes, and this area remained unchanged throughout fruit expansion (data not shown), while transpiration rates declined concurrently (Figure 4). This suggests that cranberry fruit transpiration is not modulated by stomata density or conductance. Moreover, ESEM image analysis revealed no visible evidence of wax covering the stomata (data not shown), unlike observations in highbush blueberry (Yang et al., 2020). Our greenhouse study, which involved applying vacuum grease to the calyx lobes to seal the stomata of developing fruits, resulted in minimal variation in fruit transpiration rates compared to the control fruits (Table 3). This further supports our hypothesis that cranberry fruit transpiration is not modulated by stomata density or conductance but by changes in the epidermis throughout development.

The cuticle is the outermost layer of the fruit, embedded in the epidermis, serving to protect the fruit from desiccation and radiation, among other functions (Yang et al., 2020). Water loss through the epidermis has been recognized as the primary mechanism of transpiration, with increases in cuticle thickness linked to significant reductions in transpiration rates recorded during fruit growth (Higuchi and Sakuratani, 2006; Montanaro et al., 2015; Morandi et al., 2012; Palliotti and Cartechini, 2001; Winkler et al., 2021). In our study, the increase in cuticle thickness exhibited a negative relationship with fruit transpiration (Table 9). The timing of cuticle thickening coincided with declining rates of fruit transpiration (Figures 5 and 4, respectively), a phenomenon also reported in kiwifruit and grape (Celano et al., 2009; Rogiers et al., 2004). Cuticle deposition in the cranberry fruit surface occurs rapidly during the early stages of development, similar to strawberry (Lara et al., 2004), but it tapers off as the fruit expands. This decline may lead to the formation of microcracks, which can increase water permeability in the cuticle due to elevated surface strain from rapid fruit expansion (Lara et al., 2004). Conversely, the cuticle thickness in cranberry fruits continues to increase until harvest (Figure 4), reaching thickness values similar to those reported in previous cranberry studies (Özgen et al., 2002; Stevens, 1932). This continuous cuticle deposition may enhance the fruit's impermeability to water, as no microcracks are observed on the cuticle surface. Furthermore, cranberry fruits can remain submerged in water for up to 24 h during the wet harvest without compromising fruit quality (Özgen et al., 2002).

**TABLE 9:** Multiple linear regression output of the relationship between cranberry fruit transpiration (mmol m^−2^ s^−1^) and hydraulic weighted diameter (Dh) in μm, xylem vessel density (μm^2^), and cuticle thickness (μm) through fruit development in ‘Stevens’ grown near Black River Falls in Central Wisconsin, USA.

Coefficients	Estimates	SE	t‐value	p‐value	r^2^	Adj. r^2^
Intercept ($\beta_{0}$)	10.13	0.87	11.68	0.007^a^	0.999	0.998
Cuticle thickness ($\beta_{x1}$)	−4.89	0.44	−11.18	0.008^a^		
Dh ($\beta_{x2}$)	−0.81	0.08	−9.67	0.010^a^		
Xylem vessel density ($\beta_{x3}$)	−165.12	23.34	−7.08	0.019^a^		
Cuticle thickness x Dh ($\beta_{x4}$)	0.395	0.04	9.38	0.011*		
Cuticle thickness x Xylem vessel density $(\beta_{x5}$)	79.93	11.21	7.13	0.019*		
Dh x Xylem vessel density $(\beta_{x6}$)	14.38	2.39	6.01	0.027*		
Cuticle thickness x Dh x Xylem vessel density $(\beta_{x7}$)	−6.99	1.15	−6.10	0.03*		

^a^ The p value <0.05 are significant different at α = 0.05.

Alongside the increase in cuticle thickness, in fruit crops, anatomical changes in the vascular bundles of the pedicel appear to limit water movement and Ca translocation during fruit development (Bondada et al., 2005; Choat et al., 2009; Greenspan et al., 1994; Keller et al., 2006; Rogiers et al., 2004; Song et al., 2018). In cranberry, the functionality of the pedicel xylem in both attached and detached fruits began to decline between 35 to 42 DAFB during color development, gradually progressing until no dye was detected in the fruit (Figure 6 and Table 6). The decline in pedicel xylem functionality in cranberry may be influenced by a decrease in the xylem vessel diameter and an increase in vessel density throughout fruit development, particularly in the distal portion of the pedicel. This has also been described in other species with smaller vessel diameters and higher densities than cranberry increase water resistance, creating a “bottleneck” effect that reduces water flow into the fruit (Bondada et al., 2005; Dražeta et al., 2004; Mazzeo et al., 2013; Song et al., 2018). These anatomical changes in the xylem of the pedicel may be attributed to a gradual reduction in xylem radius, both proportionally and absolutely, due to an increase in the phloem radius and pith during fruit development (Jing et al., 2023; Lang and Ryan, 1994; Miqueloto et al., 2014; Rančić et al., 2010). In addition, comparisons between attached and detached cranberry fruits revealed that the pedicel xylem vessels of detached fruit remained functional later in the season despite anatomical changes. This suggests that leaves strongly compete with fruit for water, thus reducing water flow into the various portions of the fruit (Dražeta et al., 2004; Grimm et al., 2017; Miqueloto et al., 2014).

## 5 CONCLUSION

Ca content in cranberry fruits increases during the early stages of development and remains constant until harvest. Fruit Ca accumulation is primarily related to fruit transpiration. The decline in both fruit transpiration and water flow into the fruit can be attributed to the increase in cuticle thickness and the decrease in the xylem functionality. The gradual decline in pedicel xylem functionality may be influenced by compression from the surrounding phloem and pith tissues, which affects the diameter and density of xylem vessels. These findings underscore the importance of considering multiple physiological and anatomical parameters involved with fruit nutrient accumulation and transpiration during fruit development. Optimal fruit nutrition can positively impact fruit quality and post‐harvest life, and understanding the process of nutrient accumulation as fruits develop can assist growers in maximizing fruit calcium content by prioritizing fertilization during the early stages of fruit development. However, considering climate change's impact on cranberry production, future studies should investigate the effect of extreme weather events on fruit nutrition and quality, as well as the associated physiological and anatomical parameters.

## AUTHOR CONTRIBUTIONS

Study conception and design were completed by Amaya Atucha, Beth Ann Workmaster, and Pedro Rojas‐Barros; Methodology implementation was performed by Amaya Atucha, Beth Ann Workmaster, Jyostna Mura, Juan Zalapa, and Pedro Rojas Barros. Experiment execution and data collection were carried out by Pedro Rojas Barros and Jane Wernow. Data analysis/interpretation and manuscript writing were done by Amaya Atucha, Beth Ann Workmaster, and Pedro Rojas Barros.

## FUNDING INFORMATION

This research is based upon work supported by the National Institute of Food and Agriculture, United States Department of Agriculture, through Hatch project 1025852, administered by the College of Agricultural and Life Sciences, University of Wisconsin–Madison.

## Supporting information


**Data S1**:

## Data Availability

The data that support the findings of this study are available from the corresponding author upon reasonable request and at GitHub (https://github.com/rojasbarros/Fruit-transpiration). The digital automated code to measure the percentage of dye coverage is available through GitHub (https://github.com/rojasbarros/dye_assay).
