# Supplementary material for: Fruit Cuticle Thickness and Anatomical Changes in Pedicel Xylem Vessels Influence Fruit Transpiration and Calcium Accumulation in Cranberry Fruit
Source: Physiol Plant. 2025 Jan 10;177(1):e70036. doi: 10.1111/ppl.70036 (PMC11718430; doi:10.1111/ppl.70036)
Supplement: Supplementary file 1 — Data S1: [file PPL-177-e70036-s001.pdf]

Supporting Information

Fruit Cuticle Thickness and Anatomical Changes in Pedicel Xylem Vessels Influence Fruit Transpiration and Calcium Accumulation in Cranberry Fruit

Pedro Rojas-Barros[1], Jane Wernow[1], Beth Ann Workmaster[1], Juan Zalapa[1,2], Jyostna Mura Devi[1,2], and Amaya Atucha[1*]

[1]Department of Plant and Agroecosystem Sciences, University of Wisconsin-Madison, 1575 Linden Dr., Madison, WI 53706.

[2]USDA-ARS, Vegetable Crops Research Unit, Department of Plant and Agroecosystem Sciences, University of Wisconsin-Madison, 1575 Linden Dr., Madison, WI 53706.

*** Corresponding Author:**

Amaya Atucha,

Email: atucha@wisc.edu

Supplemental table 1. Leaf tissue analysis results from cranberry 'Stevens' grown near Black River Falls in Central Wisconsin, USA.

Tissue Analysis (Unit)	'Stevens'
N (%)	0.88
P (%)	0.09
K (%)	0.46
Ca (%)	0.71
Mg (%)	0.17
S (%)	0.08
Zn (mg/kg)	19.4
Mn (mg/kg)	141.8
B (mg/kg)	26.6
Fe (mg/kg)	164.6
Cu (mg/kg)	2.3

Supplemental table 2. Soil analysis results from cranberry ‘Stevens’ near Black River Falls in Central Wisconsin, USA.

Soil analysis	Stevens
	Location 1
pH	6.0
Phosphorus	24 ppm
Potassium	28 ppm
Organic Matter (%)	0.2%
